# Optimal Partial Pressure of Oxygen Affects Outcomes in Patients With Severe Traumatic Brain Injury

**DOI:** 10.7759/cureus.9964

**Published:** 2020-08-23

**Authors:** James Wiginton, James Brazdzionis, Tye Patchana, Ryan Dorkoski, Dan E Miulli, Raed Sweiss, Margaret Rose Wacker

**Affiliations:** 1 Neurosurgery, Riverside University Health System Medical Center, Moreno Valley, USA; 2 Environmental and Plant Science, Ohio University, Athens, USA; 3 Neurosurgery, Arrowhead Regional Medical Center, Colton, USA

**Keywords:** pao2, traumatic brain injury, tbi, brain trauma, neurosurgery, partial pressure of oxygen

## Abstract

Introduction

Severe traumatic brain injury (TBI) is a leading cause of death and disability. Not all neuronal damage occurs at the time of primary injury, but rather TBI initiates a cascade of events that leads to secondary brain injury. Oxygenation is one crucial factor in maintaining brain tissue homeostasis post-injury. We performed a retrospective review of patients admitted to a single trauma center after TBI. Statistical analysis was performed to ascertain if the measured partial pressure of oxygen (PaO₂) affected overall outcome at the time of discharge from the hospital.

Materials and Methods

Statistical analysis was performed retrospectively on patients admitted with a Glasgow Coma Scale (GCS) < 8 and a diagnosis of TBI. GCS and Glasgow Outcome Scale (GOS) were calculated from physical examination findings at the time of hospital discharge or death. Patient data were separated into two groups: those with consistently higher average PaO₂ scores (≥ 150 mmHg; n = 7) and those with lower average PaO₂ scores (< 150 mmHg; n = 8). The minimum requirement to be categorized in the consistently higher group was to have an average hospital day 1 through 5 PaO₂ value of ≥ 150 mmHg.

Results

Patients with consistent hospital Day 1 through 5 PaO₂ scores of ≥ 150 mmHg had statistically significant higher GCS scores at the end of intensive care unit (ICU)-level care or hospital discharge (mean = 12, *p* = 0.01), compared to those in group 2 with lower PaO₂ levels (mean = 7.9). There was no statistically significant difference in GOS when comparing the two groups (*p* = 0.055); however, the data did show a trend toward significance.

Discussion and Conclusion

In our study we analyzed patients diagnosed with TBI and stratified them into groups based on PaO₂ ≥ or < 150 mmHg. We demonstrate overall outcome improvement based on GCS with a trend toward improved GOS. The GCS showed statistical significance in patients with PaO₂ consistently ≥ 150 mmHg versus those in group 2 over the first five days of hospitalization.

## Introduction

Severe traumatic brain injury (TBI), defined as head injury in patients with a Glasgow Coma Scale (GCS) ≤ 8, is a leading cause of death and disability that affects approximately 5.48 million individuals per year and is the cause of death for approximately 53,000 people per year [1,2]. In the United States alone, the incidence of closed head trauma is estimated to be 500,000 per year. Of these, 10% at admission are classified as severe (GCS ≤ 8), another 10% as moderate (GCS 9-12), and the remainder as mild (GCS 13-15) [3]. The GCS score has been found to have a significant inverse correlation with the outcome following severe TBI. Those with a GCS of 3 had mortality rates ranging from 72%-78%, and those with a score of 8 had a mortality of 11%-13% [4,5]. In addition to GCS and patient morbidity/mortality, the Glasgow Outcome Scale (GOS) is a widely accepted method to compare patient outcomes for those with severe TBI [4]. The GOS ranges from scores of 1 to 5: death, persistent vegetative state (PVS), severe disability (SD) (conscious but disabled), moderate disability (MD) (disabled but independent), and good recovery (GR) [6]. The GOS is determined at the end of care to best assess patient recovery and any persistent deficits as a result of neuronal damage.

Not all neuronal damage occurs at the time of primary injury, but rather TBI initiates a cascade of events that leads to secondary brain injury [7]. Secondary brain injuries are damaging events that begin immediately after the primary impact and worsen the healing of the injured brain. Oxygenation is one crucial factor in maintaining brain tissue homeostasis post-injury. Oxygen regulation is necessary for proper mitochondrial function in aerobic glycolysis, staving off glutamate neurotoxicity, and preventing the damaging effects of reactive oxygen species [8]. Further, hyperoxemia can induce cerebral vasoconstriction causing further ischemia and injury [9].

Increased mortality and worsened short-term functional outcomes have been associated with hyperoxia within the first 24 hours of hospitalization after TBI [10]. However, strict avoidance of hyperoxemia may lead to hypoxia, and post-TBI hypoxia is associated with prolonged neuroinflammation and poor outcome. Patients with documented hypoxia (O₂ saturations < 60%) had a mortality rate of 50%, and survivors were severely disabled [11,12].

Therefore, optimizing management after the primary injury can prevent further damage and improve overall outcome. Management entails close observation, typically in the intensive care unit (ICU), and requires intubation for airway protection and artificial respiration as needed. At a minimum, arterial blood gas (ABG) analysis should be performed daily with modulation of respiratory values to optimize the patient’s overall medical condition and to positively influence recovery and survival. Determining the optimal partial pressure of oxygen (PaO₂) may improve outcomes in patients with severe TBI to reduce the damage from both hyperoxemia and hypoxia.

Here we perform a retrospective review of patients admitted to a single trauma center after TBI who required intubation and subsequent monitoring in the ICU. Analysis of their ABGs was performed to ascertain if the measured PaO₂ affected overall outcome at the time of discharge from the hospital or end of medical care.

## Materials and methods

Study design

Institutional review board approval was obtained. Patients’ charts who had been admitted to Arrowhead Regional Medical Center (Colton, California) were reviewed using retrospective cohort study.

Study population

Eligible patients presented to Arrowhead Regional Medical Center with the diagnosis of severe TBI as defined by GCS ≤ 8. Age was ≥ 18. Patients were excluded if inciting trauma included lung trauma or previous diagnosis of asthma or chronic obstructive pulmonary disease (COPD).

Data sources

Various sources of data were used. These databases include the Arrowhead Regional Medical Center Neurosurgery Registry, electronic medical records, and scanned medical records.

Data abstracted

The following was abstracted from the above-described data sources: patient demographics including age and sex, past medical history, and clinical examination findings including presenting GCS and post-treatment GCS admission date, days in the ICU, and days until discharge or death. The GOS was calculated from physical examination findings at the time of discharge or death. Laboratory and monitoring data were obtained and included PaO₂.

Statistical analysis

Statistical analysis and plot construction were done using Microsoft Excel (2016) with the Analysis ToolPak, (Microsoft Corporation, Redmond, Washington). Patient data were separated into two groups: those with consistently higher PaO₂ scores (≥ 150 mmHg; n = 7) and those with consistently lower PaO₂ scores (< 150 mmHg; n = 8). The minimum requirement to be categorized in the consistently higher group was to have an average hospital Day 1 through 5 PaO₂ value of ≥ 150 mmHg (this calculation includes both minimum and maximum values). Those categorized in the consistently lower PaO₂ group conversely had average hospital Day 1 through 5 PaO₂ values of < 150 mmHg. Student's t-test was used to find potential statistical differences between groups, including correlation with GCS on presentation and discharge/end of medical care GCS and GOS.

## Results

A total of 15 patients were included in the study population from 2017 to 2019. Average GCS on arrival was 5 and at the time of discharge was 9.8 (discharge GCS ranging from 3T to 15) (“T” denotes patients with endotracheal tube or tracheostomy in place). The average GOS was 2.9. Average days in the ICU were 32.7, and average days from admission until discharge or death were 33.8.

Analysis was performed to ascertain optimal average PaO₂ for the cohort that corresponded with those patients who had a high GOS (given high GOS corresponding with better clinical outcome). It was found that a PaO₂ value of ≥ 150 mmHg during day 1 through 5 correlated with a statically significantly higher GCS at discharge or death compared to patients with a day 1 through 5 average PaO₂ of < 150 mmHg (*p* < 0.01).

Patient data (15 patients, labeled A-O) were separated into those with consistently high PaO₂s (≥ 150 mmHg; patients A, D, F, H, K, J, and L) compared to those with consistently lower PaO₂ scores (< 150 mmHg; patients N, G, C, E, B, I, O, and M) (Table 1). The minimum requirement to be included into the consistently high PaO₂ group was having an average ≥ 150 mmHg PaO₂ for hospital Days 1 through 5. There were seven patients in the consistently high PaO₂ category (top of Table 1) and eight patients in the consistently low PaO₂ category (bottom of Table 1). Initial and discharge GCS as well as GOS data are displayed in Table 2. Graphical display of the data tabulated for each patient A through O is shown in the Appendix.

**Table 1:** Comparison of PaO₂ values of patients with consistently high PaO₂ versus those with consistently low PaO₂ Min = minimum Max = maximum

		Day 1 PaO2		Day 2 PaO2		Day 3 PaO2		Day 4 PaO2		Day 5 PaO2	
Group	Patient	Min	Max	Min	Max	Min	Max	Min	Max	Min	Max
Consistently High PaO2 (>150 mmHg)	A	70	115	135	151	187	204	137	193	151	165
	D	150	243	150	195	139	175	139	179	183	191
	F	68	196	143	183	165	180	147	175	51	220
	H	124	387	156	167	139	139	117	151	129	130
	K	167	167	97	216	142	142	174	224	168	168
	J	226	278	186	236	236	236	50	71	67	110
	L	204	206	155	252	150	154	146	146	163	163
Consistently Low PaO2 (<150 mmHg)	B	106	163	134	134	145	145	110	146	82	101
	C	99	128	81	97	99	151	66	203	132	132
	E	153	153	113	113	111	111	105	105	113	144
	G	116	116	85	112	77	144	57	72	64	165
	I	108	108	144	195	94	108	133	145	105	136
	M	128	128	137	165	155	186	176	176	107	107
	N	141	182	88	88	72	72	83	83	85	85
	O	155	155	116	116	139	139	95	151	133	150

**Table 2:** Patients presenting GCS and GCS at discharge or end of care along with GOS at discharge or end of care “T” denotes patient with endotracheal tube or tracheostomy in place. GCS = Glasgow Coma Scale GOS = Glasgow Outcome Scale

Group	Patient	Presenting GCS	GCS at Discharge or End of Care	GOS
Consistently High PaO₂ (>150 mmHg)	A	3T	15	4
	D	5T	15	4
	F	6T	10T	3
	H	4T	10T	3
	K	6T	9T	3
	J	6T	15	4
	L	3T	10T	2
Consistently Low PaO₂ (>150 mmHg)	B	4T	10T	3
	C	3T	3T	2
	E	6T	9	3
	G	8T	3	1
	I	4T	6T	3
	M	3T	11T	3
	N	8T	11T	3
	O	6T	10T	3
	Average	5.0	9.8	2.9

**Figure 1:**
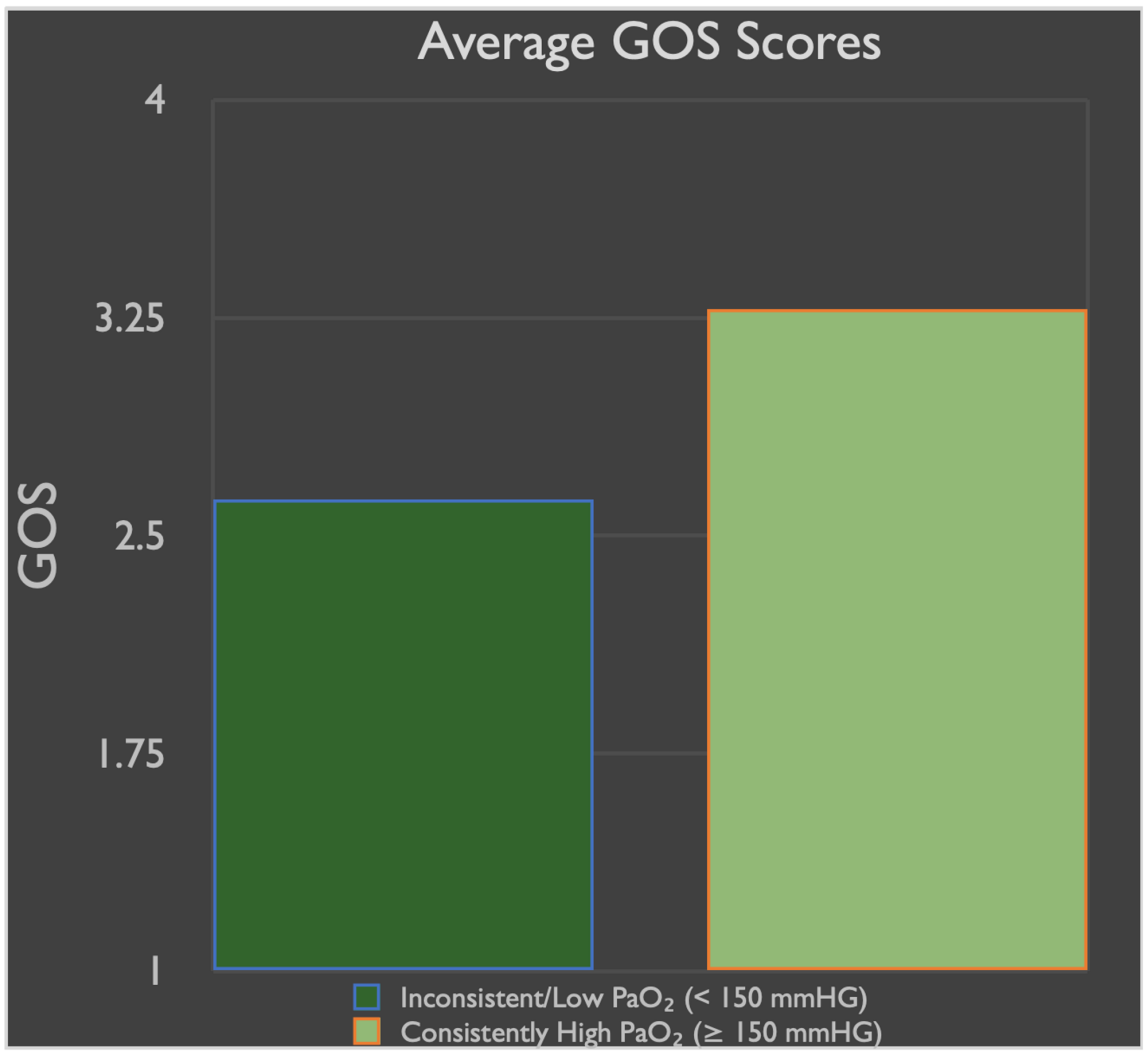
Average GOS at discharge/end of care for patients stratified into the two groups

**Figure 2:**
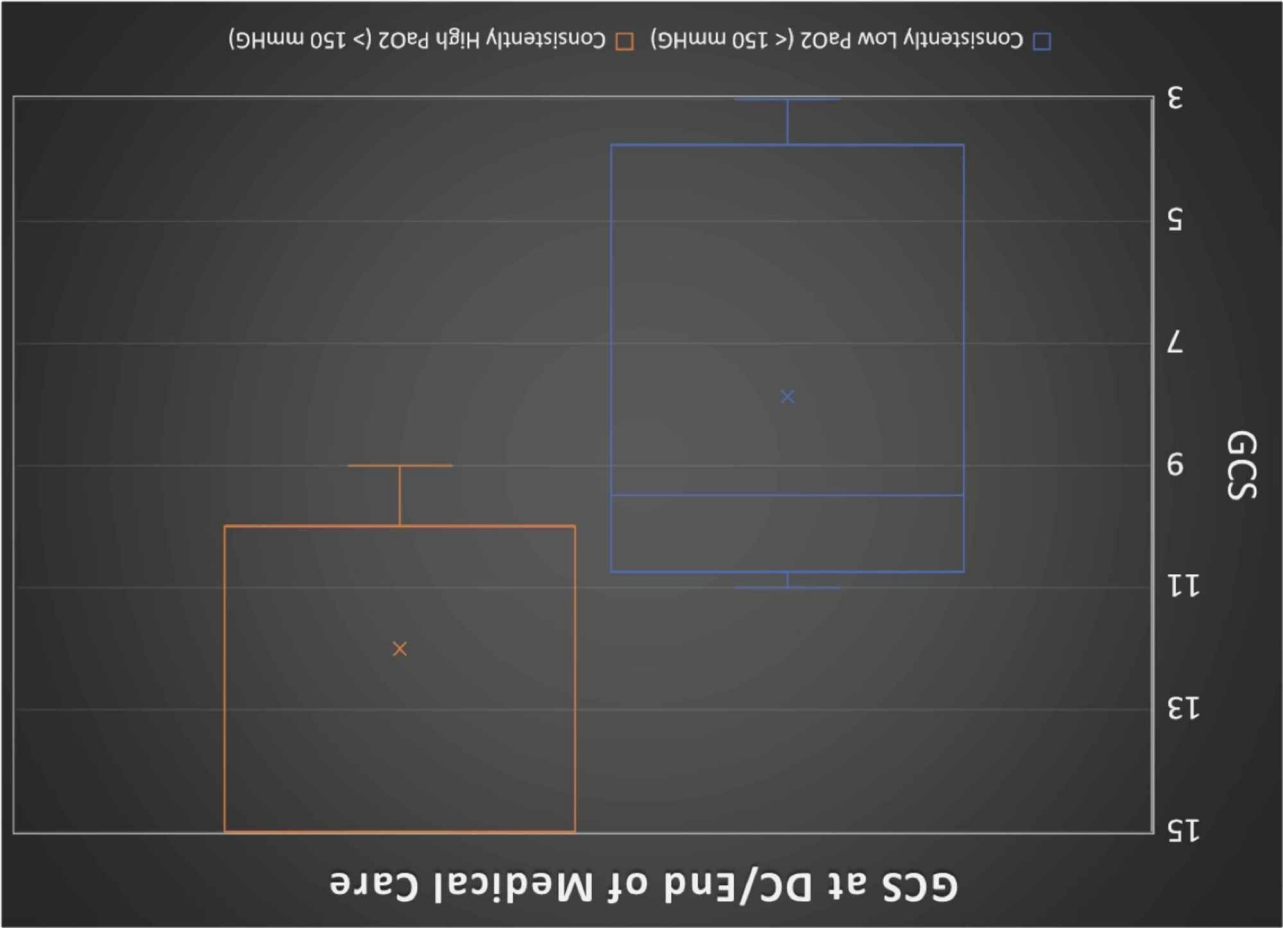
Average GCS at DC/end of care for patients stratified into the two groups GCS = Glasgow Coma Scale DC = discharge

Those with consistent hospital day 1 through 5 PaO₂ scores of ≥ 150 mmHg had statistically significantly higher GCS scores at the end of ICU-level care or discharge (p = 0.01; 48% increase). In fact, those in the consistently high averaged PaO₂ group had a mean GCS of 12 compared to GCS of 7.9 in those with lower averaged PaO₂ (Figure 1). There was no statistically significant difference in GOS when comparing the two groups (p = 0.055); however, the data did show a trend toward significance (Figure 2).

## Discussion

Brain oxygenation in TBI is important to optimize to minimize secondary injury and ultimately reduce morbidity and mortality. Arterial oxygenation in the blood, as measured by PaO₂, must be adequate to allow proper perfusion and oxygen transport to the brain. It has been shown that hypoxia in the setting of TBI can lead to overall worse outcomes [11,12].

At our institution, we have considered a PaO₂ of > 100 mmHg as measured by arterial blood gas (ABG) to be adequate in optimizing the brain’s ability to avoid secondary injury. In this study, by collecting average PaO₂ data in patients diagnosed with TBI and by correlating that data with outcomes in terms of GCS and GOS, we were able to ascertain if another PaO₂ value would be more beneficial.

Based on our available data and number of enrolled patients, we placed them into two separate groups established by post-hoc analysis: the first group with a consistent PaO₂ ≥ 150 mmHg and the second group that had a consistent PaO₂ < 150 mmHg. Using these cutoffs we compared overall GCS and GOS to determine if stratification into these groups led to an overall improvement in outcomes, or if there were no differences.

Glasgow Outcome Scale was technically not significantly higher in patients with consistent PaO₂s ≥ 150 mmHg (*p* = 0.055); however, the data showed a trend toward statistical significance and showed promising results in relation to outcome. On the other hand, a patient’s GCS at the time of discharge was statistically significantly different (*p* = 0.01) in the PaO₂ ≥ 150 mmHg group. The latter would indicate a level of correlation of blood oxygenation and improved outcomes in patients with TBI.

Of note, our study has limitations given the small sample size. While an optimal PaO₂ of ≥ 150 mmHg was found, it would be helpful to perform further research and to obtain a larger sample size. This will allow for further stratification to determine if there may be another PaO₂ value that would lead to better outcomes or if over-oxygenation/hyperoxemia would actually negatively affect outcomes. Future studies may attempt to tightly titrate PaO₂ to establish a stricter upper limit to reduce the risk of reactive oxygen damage. We also were unable to perform long-term follow-up for these patients and recognize that three-month or six-month follow-up would be ideal in terms of monitoring GOS.

Another potential limitation lies in the fact that patients with TBI typically have a multitude of comorbidities including concomitant injuries. While our exclusion criteria included known lung injury, COPD, and asthma, there could be other confounding variables that contribute to overall outcome of these patients, not least of which is their brain injury.

Despite these limitations, we believed it was important to focus on the individual aspect of blood oxygenation, since the ramifications of hypoxia can be especially severe. Hopefully, in the future there will be further studies into the individual and aggregate aspects of TBI and their influence on overall survival and outcome. Our results show that when focusing on oxygenation in TBI, a higher PaO₂ consistently ≥ 150 mmHg correlated with a statistically improved GCS at the time of discharge and a GOS trending toward positive significance.

## Conclusions

Intracranial pressure, blood pressure optimization, cerebral oxygenation, and brain tissue oxygenation are all important areas of assessment and modulation in patients with TBI. Another important aspect is blood oxygenation whereby the brain receives this necessary component and whereby secondary injury can be mitigated. In our study we analyzed patients diagnosed with TBI and stratified them into groups based on PaO₂ values ≥ or < 150 mmHg. We demonstrate overall improvement of GCS and GOS with GCS showing statistical significance in patients with PaO₂ consistently ≥ 150 mmHg over the first five days of hospitalization. Future studies with a larger sample size may help to better delineate specific thresholds including an upper limit and to analyze other aspects of this challenging disease.

## Figures and Tables

**Figure 3:**
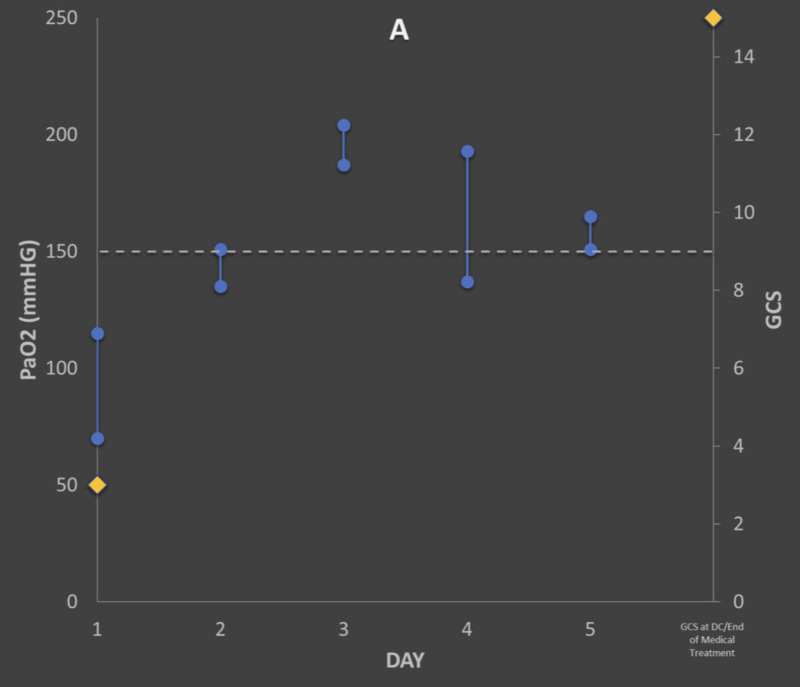
Graph for Patient A, demonstrating maximum and minimum PaO₂ levels above the predefined 150 mmHg threshold (dotted line) GCS during the first day of monitoring and at the hospital discharge or the end of ICU-level care are demonstrated by yellow diamonds. Partial pressure of oxygen (PaO₂); Glasgow Coma Scale (GCS); intensive care unit (ICU).

**Figure 4:**
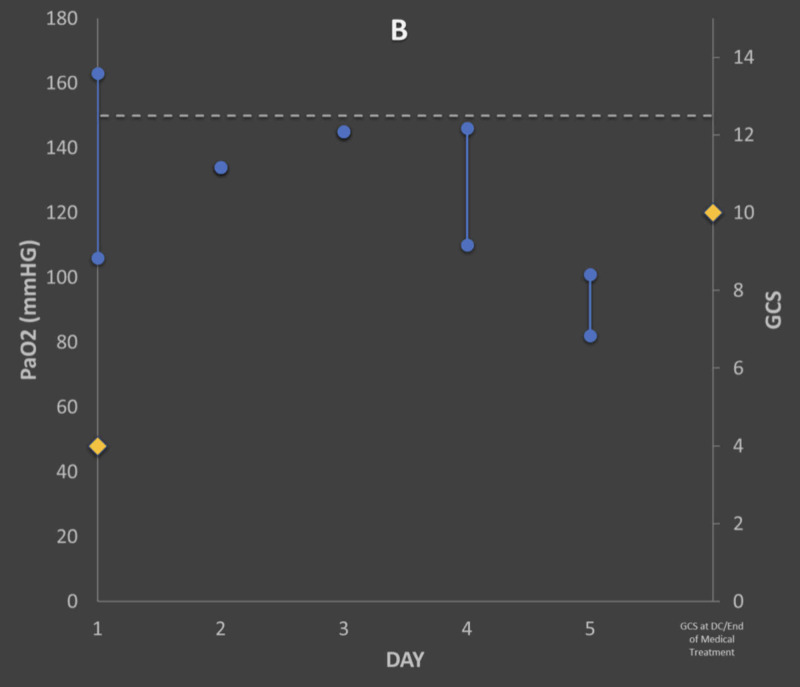
Graph for Patient B, demonstrating maximum and minimum PaO₂ levels above the predefined 150 mmHg threshold (dotted line) GCS during the first day of monitoring and at the hospital discharge or the end of ICU-level care are demonstrated by yellow diamonds. Partial pressure of oxygen (PaO₂); Glasgow Coma Scale (GCS); intensive care unit (ICU).

**Figure 5:**
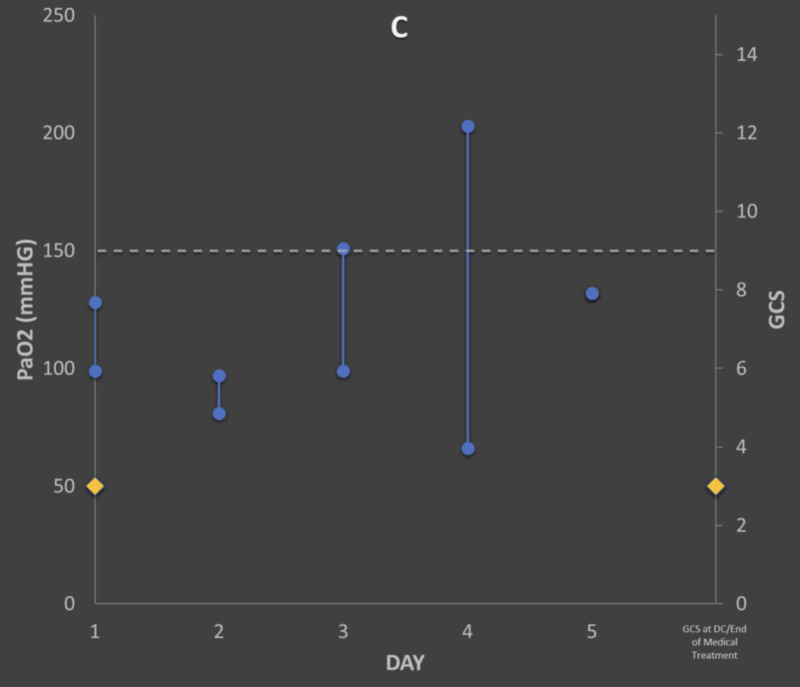
Graph for Patient C, demonstrating maximum and minimum PaO₂ levels above the predefined 150 mmHg threshold (dotted line) GCS during the first day of monitoring and at the hospital discharge or the end of ICU-level care are demonstrated by yellow diamonds. Partial pressure of oxygen (PaO₂); Glasgow Coma Scale (GCS); intensive care unit (ICU).

**Figure 6:**
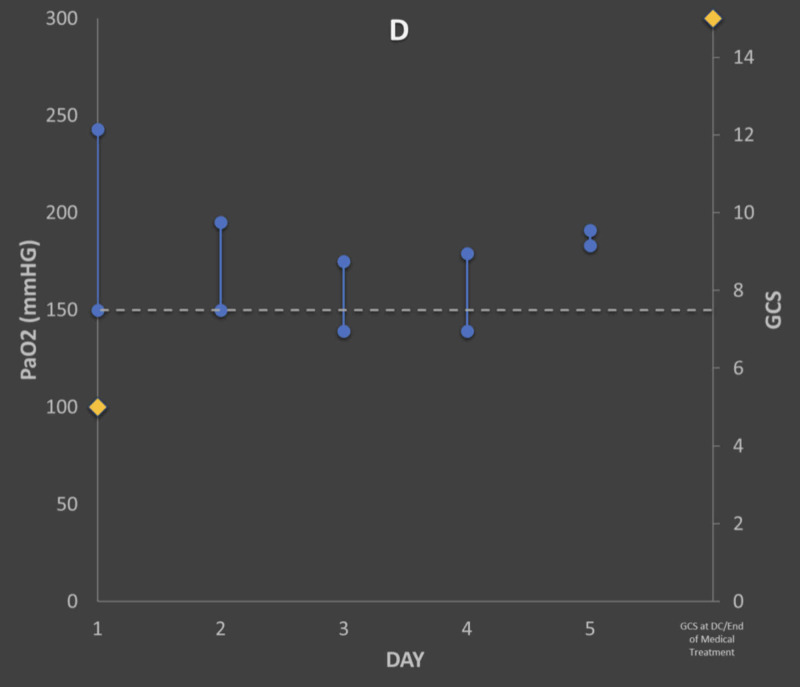
Graph for Patient D, demonstrating maximum and minimum PaO₂ levels above the predefined 150 mmHg threshold (dotted line) GCS during the first day of monitoring and at the hospital discharge or the end of ICU-level care are demonstrated by yellow diamonds. Partial pressure of oxygen (PaO₂); Glasgow Coma Scale (GCS); intensive care unit (ICU).

**Figure 7:**
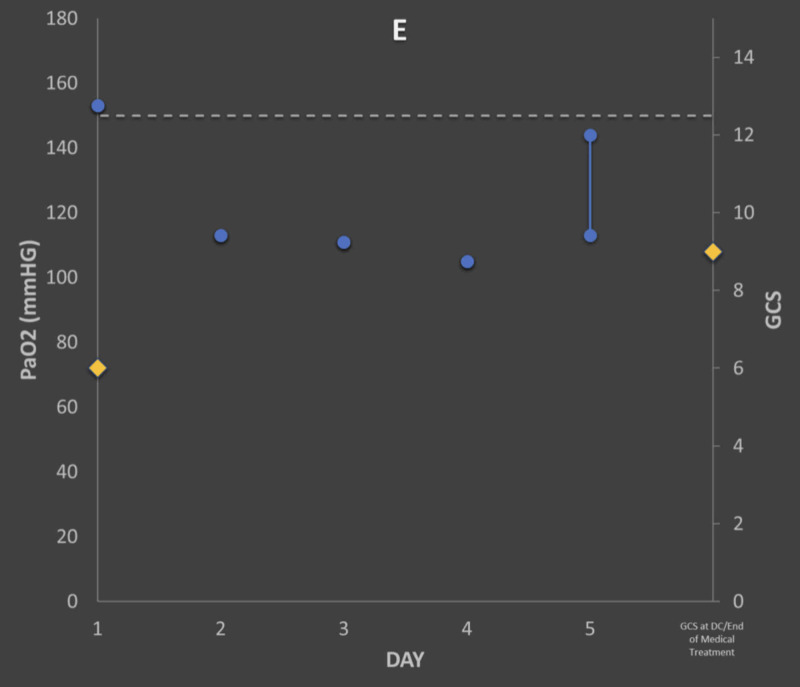
Graph for Patient E, demonstrating maximum and minimum PaO₂ levels above the predefined 150 mmHg threshold (dotted line) GCS during the first day of monitoring and at the hospital discharge or the end of ICU-level care are demonstrated by yellow diamonds. Partial pressure of oxygen (PaO₂); Glasgow Coma Scale (GCS); intensive care unit (ICU).

**Figure 8:**
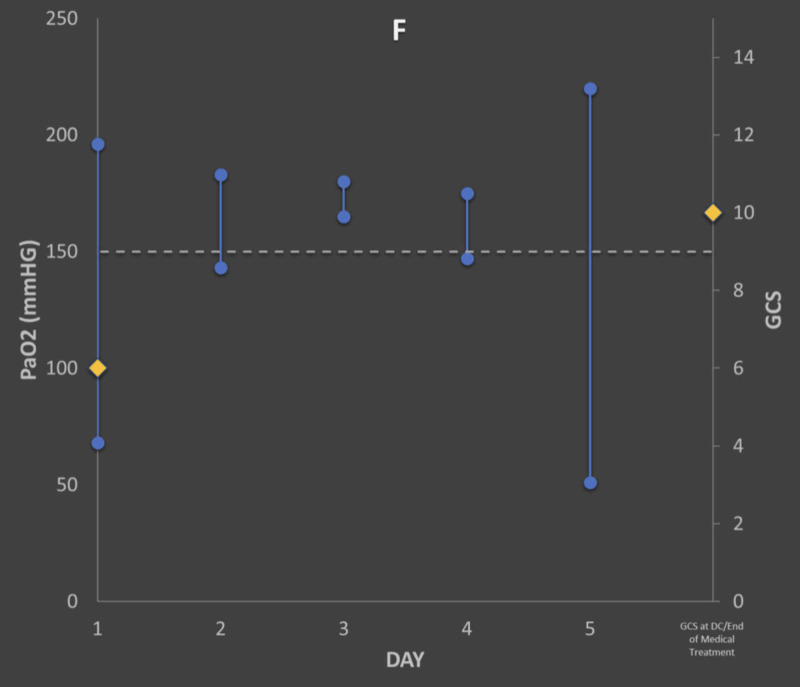
Graph for Patient F, demonstrating maximum and minimum PaO₂ levels above the predefined 150 mmHg threshold (dotted line) GCS during the first day of monitoring and at the hospital discharge or the end of ICU-level care are demonstrated by yellow diamonds. Partial pressure of oxygen (PaO₂); Glasgow Coma Scale (GCS); intensive care unit (ICU).

**Figure 9:**
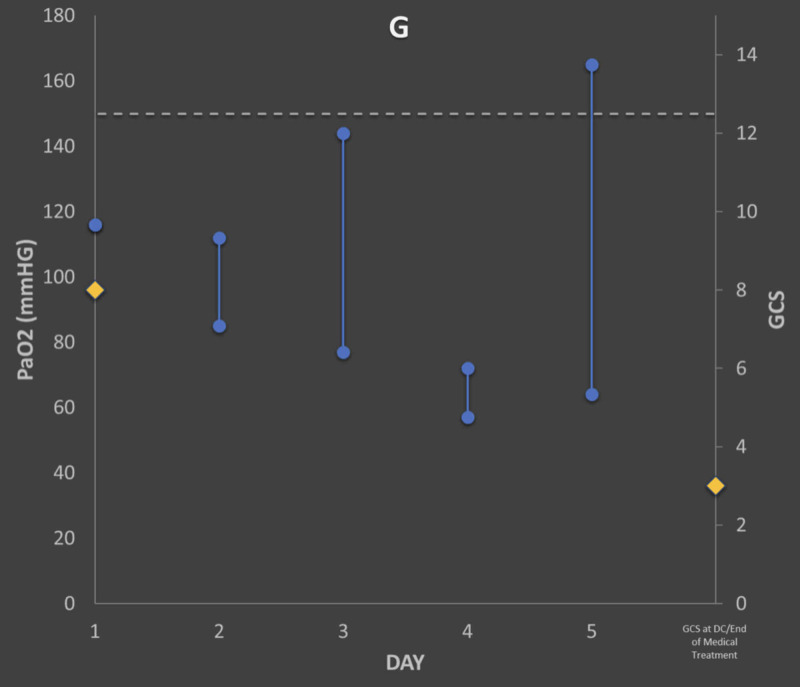
Graph for Patient G, demonstrating maximum and minimum PaO₂ levels above the predefined 150 mmHg threshold (dotted line) GCS during the first day of monitoring and at the hospital discharge or the end of ICU-level care are demonstrated by yellow diamonds. Partial pressure of oxygen (PaO₂); Glasgow Coma Scale (GCS); intensive care unit (ICU).

**Figure 10:**
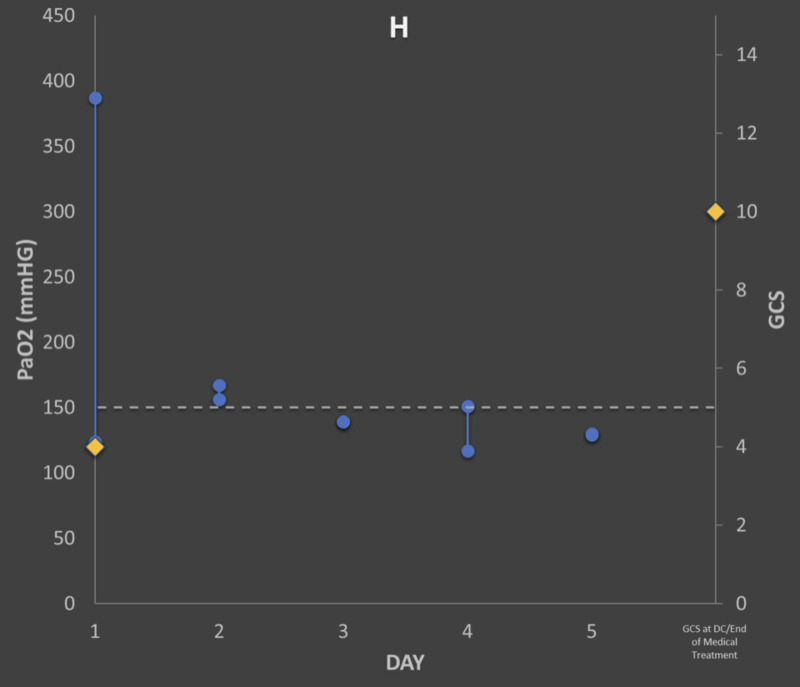
Graph for Patient H, demonstrating maximum and minimum PaO₂ levels above the predefined 150 mmHg threshold (dotted line) GCS during the first day of monitoring and at the hospital discharge or the end of ICU-level care are demonstrated by yellow diamonds. Partial pressure of oxygen (PaO₂); Glasgow Coma Scale (GCS); intensive care unit (ICU).

**Figure 11:**
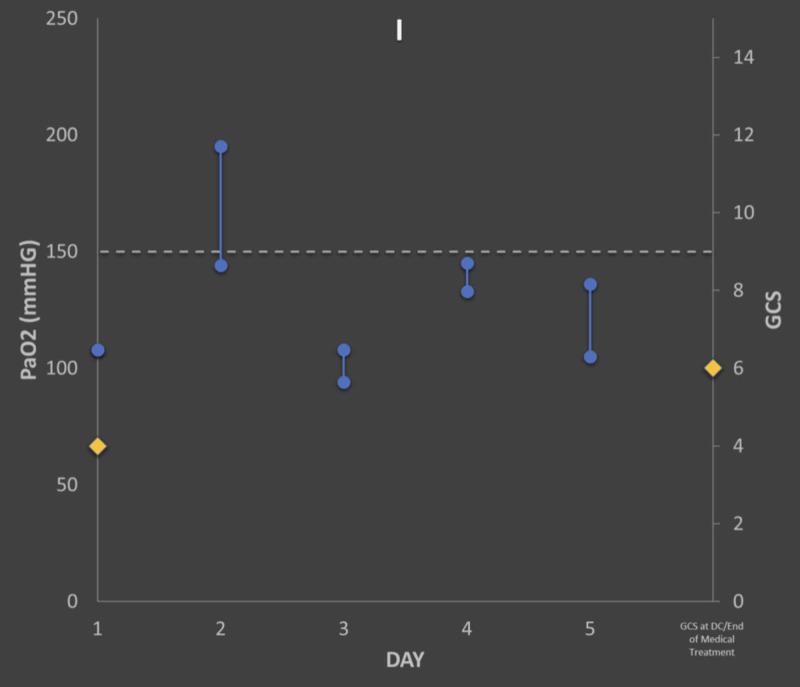
Graph for Patient I, demonstrating maximum and minimum PaO₂ levels above the predefined 150 mmHg threshold (dotted line) GCS during the first day of monitoring and at the hospital discharge or the end of ICU-level care are demonstrated by yellow diamonds. Partial pressure of oxygen (PaO₂); Glasgow Coma Scale (GCS); intensive care unit (ICU).

**Figure 12:**
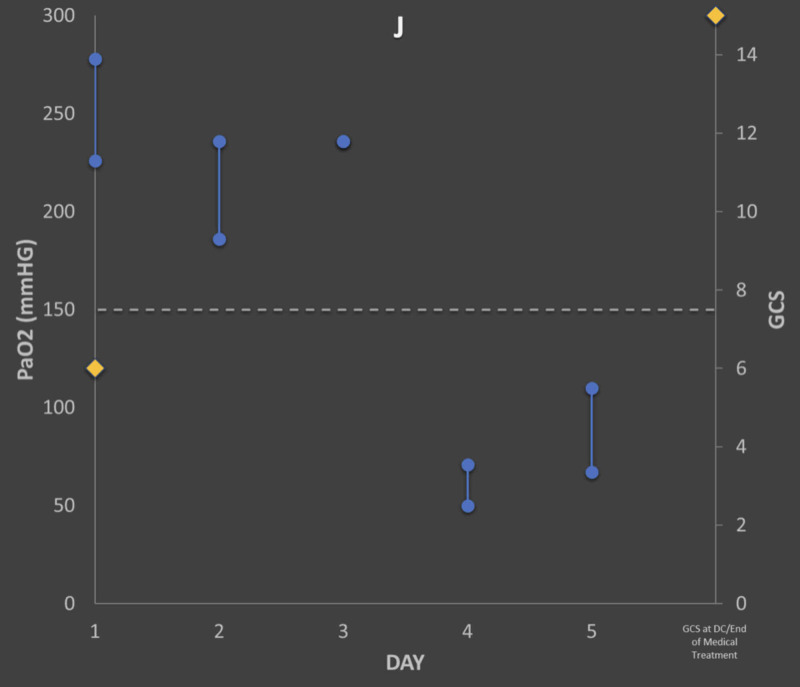
Graph for Patient J, demonstrating maximum and minimum PaO₂ levels above the predefined 150 mmHg threshold (dotted line) GCS during the first day of monitoring and at the hospital discharge or the end of ICU-level care are demonstrated by yellow diamonds. Partial pressure of oxygen (PaO₂); Glasgow Coma Scale (GCS); intensive care unit (ICU).

**Figure 13:**
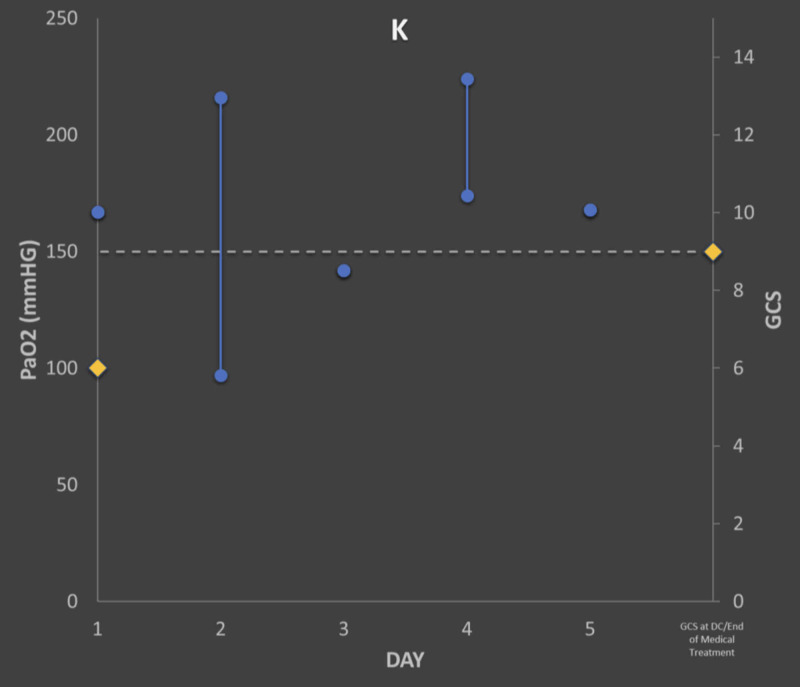
Graph for Patient K, demonstrating maximum and minimum PaO₂ levels above the predefined 150 mmHg threshold (dotted line) GCS during the first day of monitoring and at the hospital discharge or the end of ICU-level care are demonstrated by yellow diamonds. Partial pressure of oxygen (PaO₂); Glasgow Coma Scale (GCS); intensive care unit (ICU).

**Figure 14:**
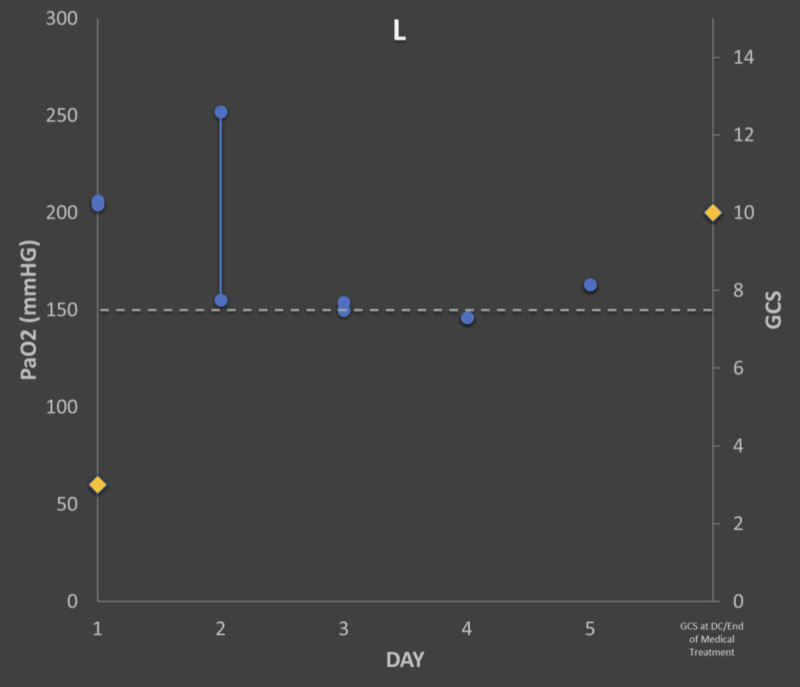
Graph for Patient L, demonstrating maximum and minimum PaO₂ levels above the predefined 150 mmHg threshold (dotted line) GCS during the first day of monitoring and at the hospital discharge or the end of ICU-level care are demonstrated by yellow diamonds. Partial pressure of oxygen (PaO₂); Glasgow Coma Scale (GCS); intensive care unit (ICU).

**Figure 15:**
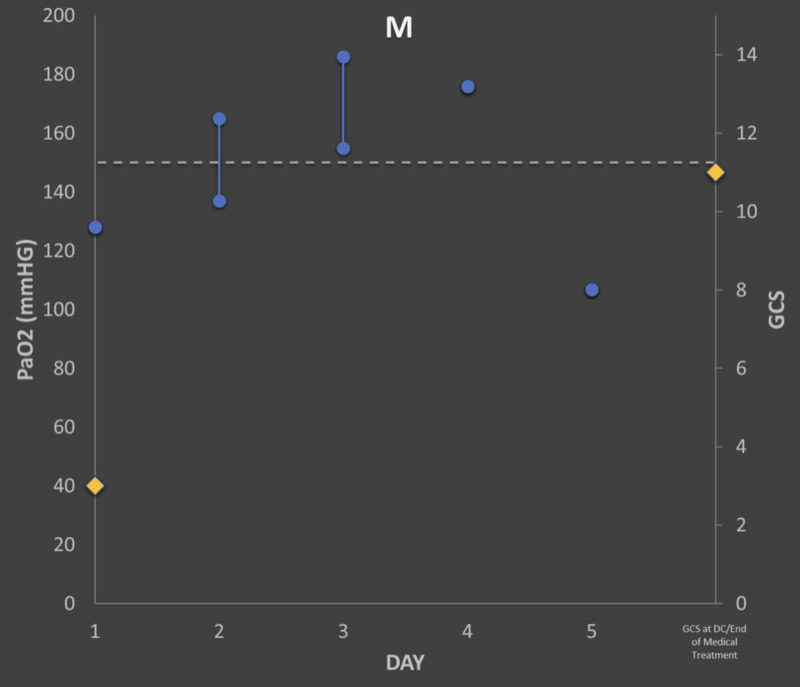
Graph for Patient M, demonstrating maximum and minimum PaO₂ levels above the predefined 150 mmHg threshold (dotted line) GCS during the first day of monitoring and at the hospital discharge or the end of ICU-level care are demonstrated by yellow diamonds. Partial pressure of oxygen (PaO₂); Glasgow Coma Scale (GCS); intensive care unit (ICU).

**Figure 16:**
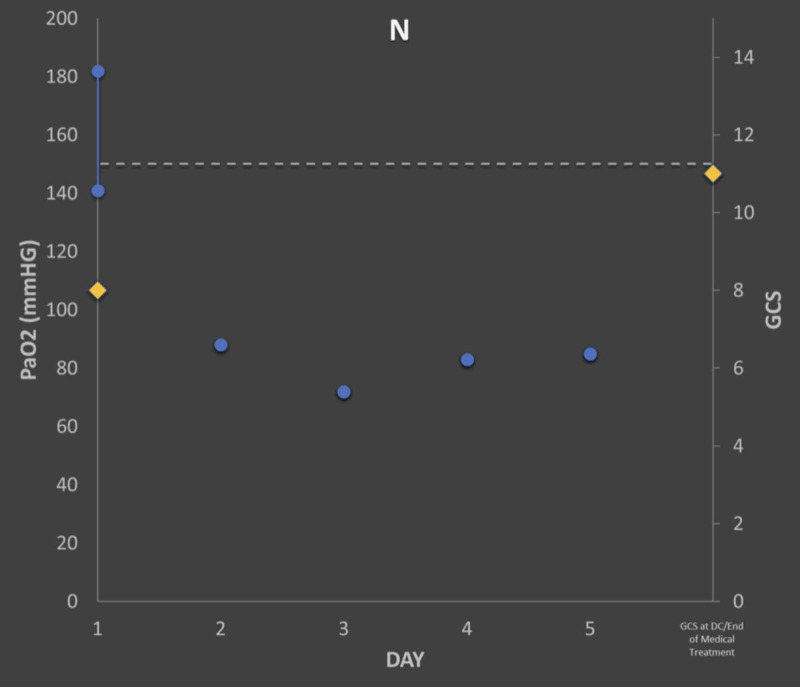
Graph for Patient N, demonstrating maximum and minimum PaO₂ levels above the predefined 150 mmHg threshold (dotted line) GCS during the first day of monitoring and at the hospital discharge or the end of ICU-level care are demonstrated by yellow diamonds. Partial pressure of oxygen (PaO₂); Glasgow Coma Scale (GCS); intensive care unit (ICU).

**Figure 17:**
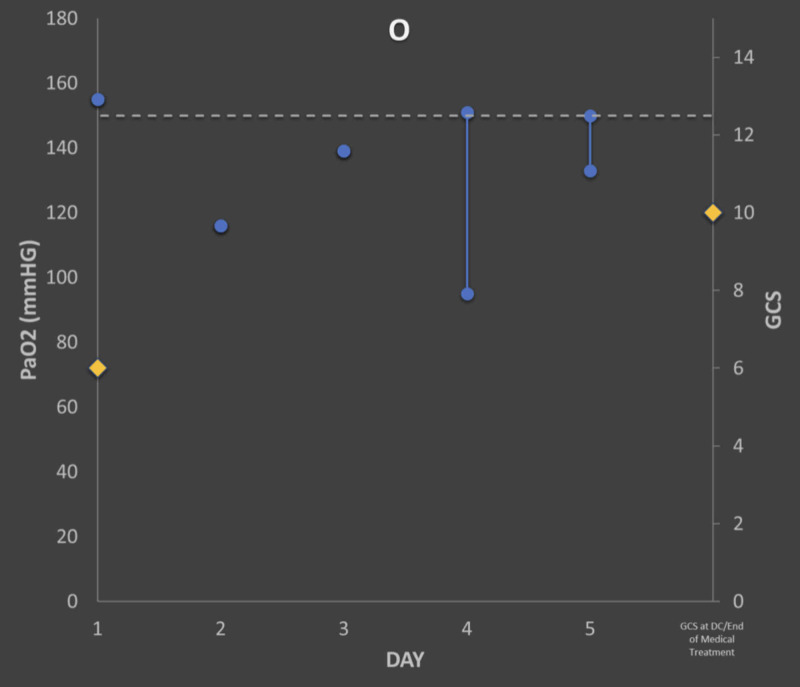
Graph for Patient O, demonstrating maximum and minimum PaO₂ levels above the predefined 150 mmHg threshold (dotted line) GCS during the first day of monitoring and at the hospital discharge or the end of ICU-level care are demonstrated by yellow diamonds. Partial pressure of oxygen (PaO₂); Glasgow Coma Scale (GCS); intensive care unit (ICU).

## References

[1] Iaccarino C, Carretta A, Nicolosi F, Morselli C (2018). Epidemiology of severe traumatic brain injury. J Neurosurg Sci.

[2] Coronado VG, Xu L, Basavaraju SV (2011). Surveillance for traumatic brain injury-related deaths--United States, 1997-2007. MMWR Surveill Summ.

[3] Narayan RK, Michel ME, Ansell B (2002). Clinical trials in head injury. J Neurotrauma.

[4] Corral L, Ventura JL, Herrero JI (2007). Improvement in GOS and GOSE scores 6 and 12 months after severe traumatic brain injury. Brain Inj.

[5] Jiang JY, Gao GY, Li WP, Yu MK, Zhu C (2002). Early indicators of prognosis in 846 cases of severe traumatic brain injury. J Neurotrauma.

[6] Jennet B, Bond M (1975). Assessment of outcome after severe brain damage: a practical scale. Lancet.

[7] Spiotta AM, Stiefel MF, Gracias VH (2010). Brain tissue oxygen-directed management and outcome in patients with severe traumatic brain injury. J Neurosurg.

[8] Enriquez P, Bullock R (2004). Molecular and cellular mechanisms in the pathophysiology of severe head injury. Curr Pharm Des.

[9] Davis DP, Meade W, Sise MJ (2009). Both hypoxemia and extreme hyperoxemia may be detrimental in patients with severe traumatic brain injury. J Neurotrauma.

[10] Brenner M, Stein D, Hu P, Kufera J, Wooford M, Scalea T (2012). Association between early hyperoxia and worse outcomes after traumatic brain injury. Arch Surg.

[11] Yan EB, Satgunaseelan L, Paul E (2014). Post-traumatic hypoxia Is associated with prolonged cerebral cytokine production, higher serum biomarker levels, and poor outcome in patients with severe traumatic brain injury. J Neurotrauma.

[12] Bratton SL, Chestnut RM, Ghajar J (2007). I. Blood pressure and oxygenation. J Neurotrauma.

